# Alternative Treatment Approach for Right Heart Masses

**DOI:** 10.7759/cureus.3673

**Published:** 2018-12-03

**Authors:** Abhishek Bhagat, Frank H Annie, Alfred Tager, Aravinda Nanjundappa, Christopher Adams

**Affiliations:** 1 Internal Medicine, Charleston Area Medical Center, Charleston, USA; 2 Cardiology, Charleston Area Medical Center, Charleston, USA; 3 Emergency Medicine, Charleston Area Medical Center, Charleston, USA; 4 Cardiology, Charleston Area Medical Center / West Virginia University, Charleston, USA

**Keywords:** pmt, heart mass

## Abstract

The traditional therapeutic approach for heart masses has been surgical resection. For right-sided masses, percutaneous mechanical thrombectomy (PMT) is a viable treatment option which is being applied with increasing frequency. This newer treatment modality is less invasive, less expensive, and results in shorter hospital stays compared to cardiac surgery. We demonstrate below a case in which rheolytic PMT was utilized successfully, allowing the patient to be discharged the following day.

## Introduction

Heart masses (abnormal growth in or adjacent to the heart) as well as opiate epidemic are increasing in frequency in aging population. These heart masses are classified into three categories: Tumor, thrombus and vegetation. Tumors are of malignant or benign etiology [1]. Thrombi can be of type A which are highly mobile and associated with deep venous thrombosis (DVT)/pulmonary embolism (PE) or type B which tend to be non-mobile and form in situ [1-4]. The third variety of heart masses is vegetation which is primarily caused by bacterial infections. These lesions are frequently identified upon workup for endocarditis, stroke, murmur, or are incidental findings. For most heart masses, the standard treatment approach has been surgical resection. For the right-sided lesions, percutaneous mechanical thrombectomy (PMT) is a viable treatment option. Primary cardiac tumors are rare (less than 0.1% of the population) [1], and papillary fibroelastomas (PFEs) account for approximately 10% of primary cardiac tumors [2-4] and the most frequent type affecting the valves. Although they do carry the potential for embolization, they are usually discovered incidentally (via echocardiography or cardiac computed tomography/magnetic resonance imaging (CT/MRI)). Despite being traditionally detected on valvular surfaces, they do not cause valvular dysfunction. Most PFEs are located in the left heart. Here we present an unusual case of a PFE of the right atrium, allowing for PMT.

## Case presentation

**Figure 1:**
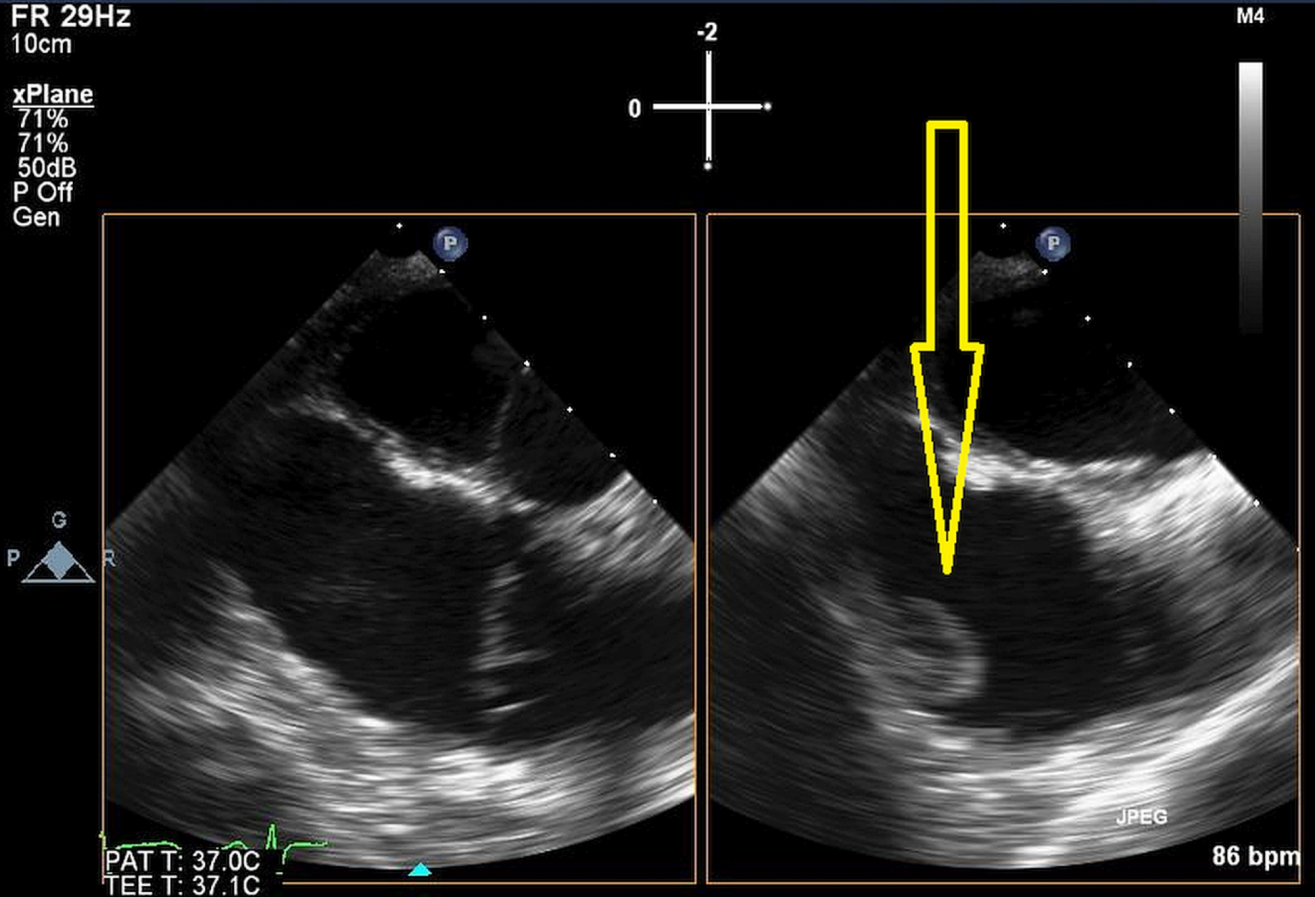
Transthoracic echocardiogram (TTE) revealed a right atrial mass measuring 1.5 cm x 2 cm. Arrow as shown displays position of right heart mass.

A 72-year-old man with past medical history significant for a stroke and hypertension was undergoing routine evaluation for his muscular dystrophy. Transthoracic echocardiogram (TTE) revealed a right atrial mass measuring 1.5 cm x 2 cm (Figure 1).

**Figure 2:**
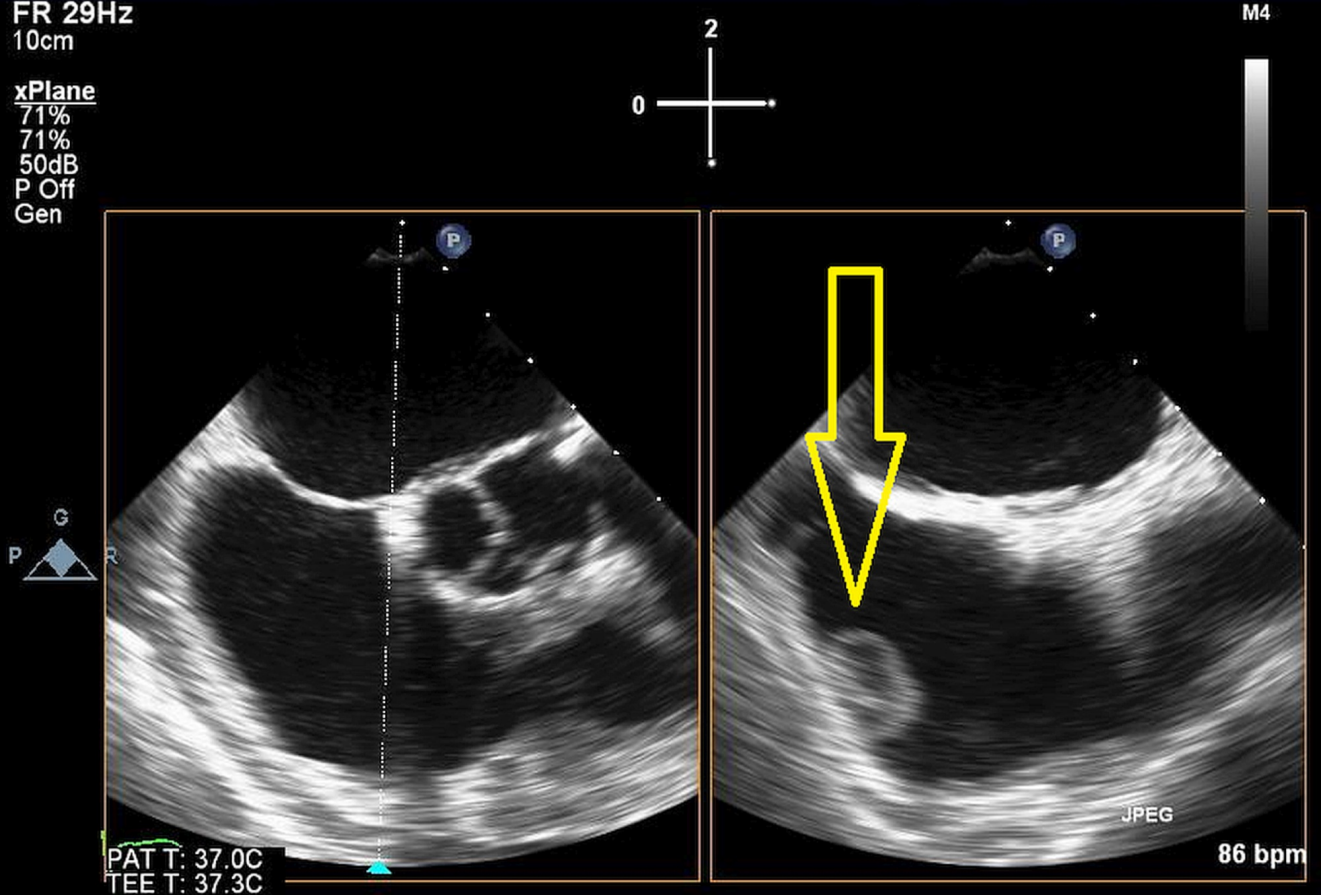
Transesophageal echocardiogram additional image. Arrow as shown displays 2D image of right heart mass and attachment onto the surrounding wall.

**Figure 3:**
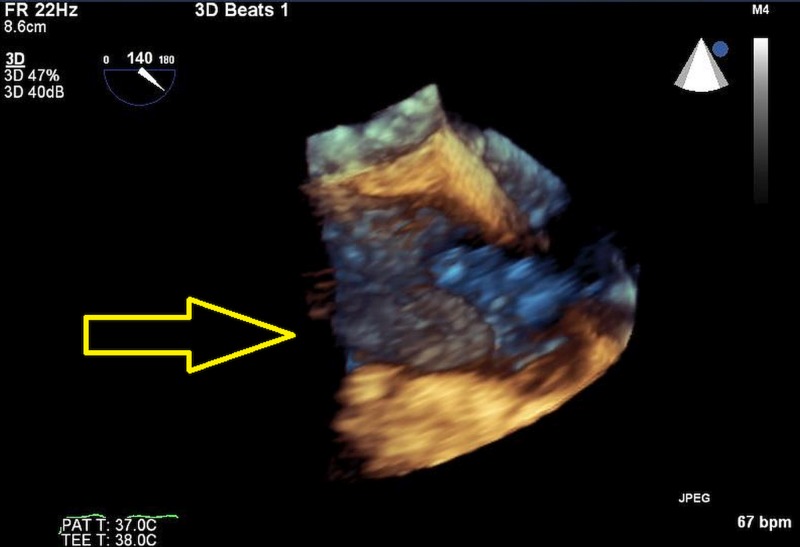
3D model of mass. Arrow displays position and size of mass related to the heart wall.

Transesophageal echocardiogram (TEE) results were consistent with this finding (Figures 2-3).

**Figure 4:**
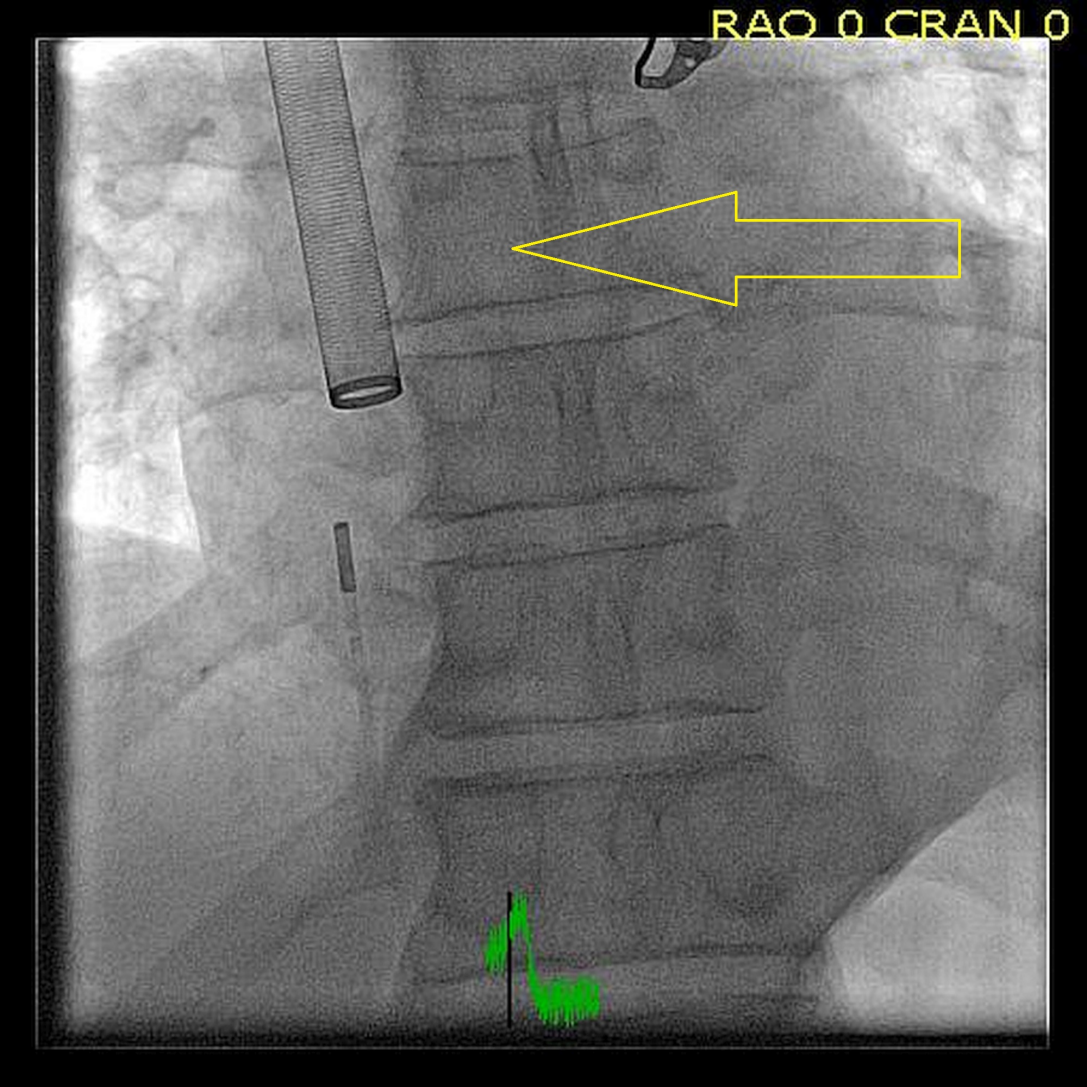
Percutaneous mechanical thrombectomy (PMT) using AngioVac. PMT being deployed to extract a right heart mass. PMT is a minimally invasive adjunctive therapy used to treat thrombosis like deep venous thrombosis (DVT) or pulmonary embolism (PE).

He was started on anticoagulation and transferred to our facility for PMT using AngioVac (Figure 4).

For this procedure, the right internal jugular vein and bilateral common femoral veins were accessed. Intracardiac echo was used. The intracardiac echo showed an approximately 1.5 cm x 2 cm mass in the right atrium. This was ultimately extracted with 0% leftover tissue burden. Pump time was approximately two minutes. 22 French AngioVac cannula had been fashioned with a heat gun. This was then introduced from the right internal jugular vein. Blood was delivered via the left common femoral vein. Intracardiac echo was performed from the right common iliac vein. Tissue was sent to pathology for further review which confirmed the diagnosis of PFE. The patient was then discharged home without the need for long-term anticoagulation.

## Discussion

The PMT has mostly been used as an adjunct therapy for the treatment of DVT. PMT is classified into rheolytic, rotational, or ultrasound-enhanced. Rheolytic PMT sprays a thrombolytic agent followed by pressurized saline at the thrombus [1]. The softened thrombus is then suctioned by the catheter into a collecting canister. Rotational devices function to macerate the thrombus via a high-velocity rotating helix [3]. Ultrasound-enhanced devices employ catheters containing ultrasound transducers which emit high-frequency, low-energy waves to expand and thin the fibrin component of the thrombus. This exposes plasminogen receptors and allows for higher penetration by the lytic agent. PMT, compared to surgical resection of cardiac masses, does not warrant a sternotomy or cardioplegic cardiac arrest – minimizing the associated complications [3]. The average hospitalization time after surgical resection of heart masses is approximately five days. In contrast, most patients are able to be discharged the following day with PMT. From a financial perspective, surgical resection costs approximately $130,000, whereas PMT costs $52,000 [1-4].

## Conclusions

Upon our review of the literature, no reports were found in which PMT was applied for right-sided heart masses (tumors, vegetations, thrombi). However, PMT is successfully being used for the treatment of massive pulmonary emboli, DVTs, and ischemic strokes. In this case, the patient was started on anticoagulants after a 1.5 cm x 2 cm right atrial mass was confirmed using TTE, TEE and intracardiac echo. The pathology report of the mass after having it totally extracted confirmed the diagnosis of PFE; therefore, the anticoagulants were discontinued. We hypothesize that PMT will lead to less morbidity and mortality, cost, and hospitalization, compared to standard surgical resection for these right-sided lesions. Further studies examining these two approaches are recommended.
